# Biosensors for Early Detection of Parkinson’s Disease: Principles, Applications, and Future Prospects

**DOI:** 10.3390/bios15050280

**Published:** 2025-04-29

**Authors:** Panpan Jiang, Nan Gao, Gang Chang, Yuxiang Wu

**Affiliations:** 1 College of Optoelectronic Materials and Technology, Jianghan University, Wuhan 430056, China; 18062575964@163.com; 2 Institute of Intelligent Sport and Proactive Health, Department of Health and Physical Education, Jianghan University, Wuhan 430056, China; gao2411@jhun.edu.cn; 3 Ministry of Education Key Laboratory for the Green Preparation and Application of Functional Materials, Hubei Key Laboratory of Polymer Materials, School of Materials Science and Engineering, Hubei University, Wuhan 430062, China; changgang@hubu.edu.cn

**Keywords:** biosensor, biomarker, early diagnosis, Parkinson’s disease

## Abstract

Parkinson’s disease (PD), a neurodegenerative disorder marked by the progressive loss of dopaminergic neurons in the substantia nigra, imposes substantial economic burdens, including both direct and indirect costs. The medical community currently lacks a definitive cure for Parkinson’s disease, and early detection is crucial for timely intervention and disease management. As innovative diagnostic tools, biosensors have shown great potential in detecting PD at its early stages. This review comprehensively summarizes recent advances in biosensors for the early detection of PD, with a particular focus on the detection of two key biomarkers: dopamine (DA) and α-synuclein (α-syn). Furthermore, it illustrates a variety of nanotechnology-based biosensors, including optical, electrochemical, and transistor biosensors, detailing their underlying principles, advantages, limitations, and applications in PD detection. Moreover, the review explores the challenges and prospects of advancing biosensors for early PD diagnosis.

## 1. Introduction

Parkinson’s disease (PD), the second most prevalent neurodegenerative disease after Alzheimer’s disease, primarily affects middle-aged and older adults [1]. Progressive loss of dopaminergic neurons in the pars compacta nigra (SNpc) is strongly associated with PD, and symptoms worsen as more dopaminergic neurons die [2,3]. Depending on the level of dopaminergic cell death, symptoms can be divided into non-motor symptoms, such as depression, hyposmia, cognitive impairment, sleep disturbances, and constipation; and motor characteristics, including bradykinesia, gait disturbance, quiescence tremor, myotonia, and speech deficits [4,5]. PD may also cause a series of complications, such as aspiration pneumonia, accumulation pneumonia, urinary tract infection, etc. These complications may further aggravate the patient’s condition. Currently, there is no cure for PD, and early intervention is crucial to slow the progression of the condition.

**Figure 1:**
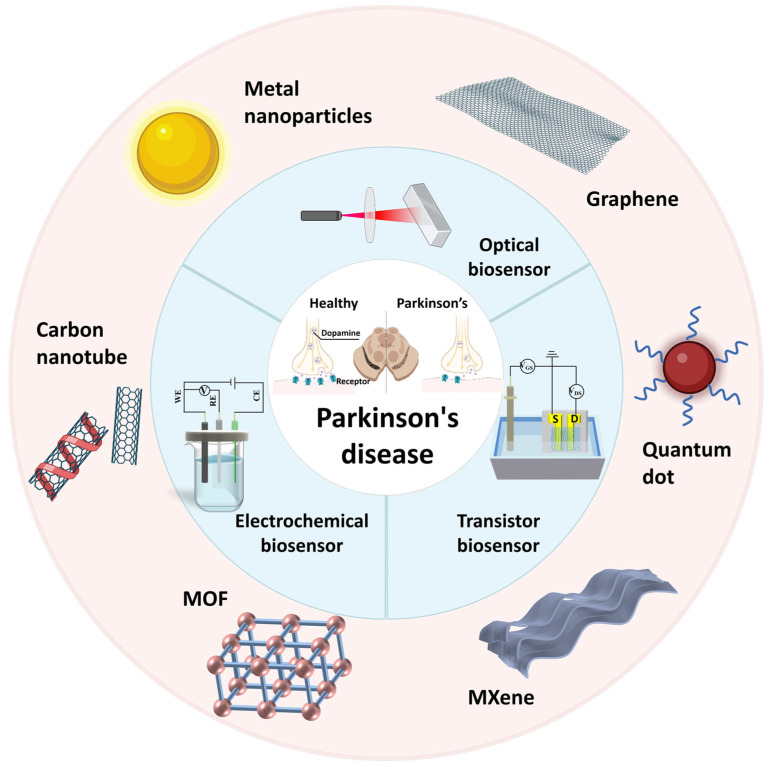
Main biosensor for Parkinson’s disease detection. (S: Source, D: Drain, MOF: Metal-Organic Framework, it consists of metal nodes—the spheres in the figure—and organic ligands).

Early detection of PD is crucial for timely intervention and disease management, as it allows for the initiation of appropriate treatments and interventions that can potentially improve patient outcomes. Traditional diagnostic methods for PD rely on clinical observation and subjective assessment, which may lead to misdiagnosis and delayed treatment initiation. The advantages of biosensors, including their simple structure, low cost, high availability, and ease of implementation and interpretation of results, make them a promising alternative method for early detection of PD [6]. Conventional biosensors, however, exhibit certain limitations including restricted sensitivity, challenges in detecting target molecules at low concentrations, and limited anti-interference capability. In recent years, nanotechnology-based biosensors have shown promise in the early detection of PD (Figure 1). These biosensors utilize the unique properties of biomarkers associated with PD to detect and quantify their presence in various biological samples. Increased performance of biosensors was achieved with high surface area-conducting nanomaterials, such as Au-based materials, nanostructures, graphene-related materials, etc. [7]. They offer improved sensitivity and selectivity in detecting PD-related molecules in biological samples.

In this review, recent advances in biosensors for the early detection of Parkinson’s disease (PD) are presented. The operational principles of various nanotechnology-based biosensors (optical, electrochemical, and transistors) and their potential applications in clinical settings are summarized. Furthermore, we discuss the opportunities and challenges of biosensors for early detection of PD.

## 2. Parkinson’s Disease Biomarkers

Biomarkers serve as a significant indicator of human quality of life and are often closely associated with disease pathology [8]. Dopaminergic neuron loss occurs gradually in PD, a complicated neurological illness. The pathological mechanisms of this disease involve multiple biomarkers, the most prominent of which are dopamine (DA) and α-synuclein.

Dopamine plays an important role in almost all cognitive functions, including motor control, motivation, and learning. In mammals, DA accounts for 80% of the catecholamine content in the brain and is a major neurotransmitter [9]. The densest area of dopamine innervation is the striatum, a large subcortical region with multiple functional branches that is the center of the large basal ganglia circuit [9]. The main pathophysiological features of PD are based on the selective loss of dopamine neurons in the substantia nigra striatum, especially those located in the lateral substantia nigra [9]. In most investigations, the cerebrospinal fluid (CSF) of untreated patients with PD exhibited significantly reduced levels of DA, indicating a loss of dopaminergic cells. A decline in DA levels ultimately leads to the manifestation of motor symptoms, such as bradykinesia, rigidity, tremor, and postural instability. These findings suggest that quantifying dopamine (DA) levels could serve as a valuable diagnostic tool for identifying early-stage nigrostriatal dopaminergic denervation. Consequently, the approach could facilitate the development of therapeutic strategies aimed at slowing or potentially halting disease progression [10].

α-synuclein (α-Syn) is a 14 kDa protein consisting of 140 amino acids, which is encoded by the SNCA gene located on chromosome 4 [2]. The major variant (α-syn-140) can be structurally divided into two distinct functional regions, namely the N- and C-termini [3]. The N-terminal amphipathic region undergoes conformational changes, forming an extended helix upon binding to negatively charged phospholipid vesicles, while a broken helix is formed when binding to phospholipid micelles [11]. The C-terminal of α-syn is characterized by its acidity, high glutamate content, and persistent disorderliness even in the presence of a membrane [11]. Post-translational modifications and cellular stress are believed to initiate the misfolding of α-syn, leading to its subsequent aggregation into toxic oligomers and fibrils [12]. The α-syn protein is primarily expressed in the brain at presynaptic terminals. It is believed to play a significant role in facilitating the mobilization of synaptic vesicles for the exocytotic release of neurotransmitters into the synaptic cleft [3]. Although α-syn predominantly resides intracellularly, it has also been detected in extracellular fluids such as cerebrospinal fluid, blood, and plasma [3]. The biosensor mainly detects α-syn in extracellular fluid.

In recent years, research on biomarkers in Parkinson’s disease has expanded significantly. Emerging evidence highlights the diagnostic and prognostic potential of other biomarkers associated with PD pathophysiology beyond α-synuclein and dopamine, including glucocerebrosidase (GCase) [13,14,15], glial fibrillary acidic protein (GFAP) [16,17], neurofilament light chain (NfL) [18], DJ-1 [19,20], and others [21]. Glucocerebrosidase (GCase), a lysosomal enzyme encoded by the GBA1 gene, catalyzes the hydrolysis of glucocerebroside into glucose and ceramide [15,22]. Dysregulation of lysosomal function disrupts lipid metabolism, resulting in toxic substrate accumulation [22]. Specifically, diminished GCase activity induces glycosphingolipid retention, lysosomal–autophagic dysfunction, and subsequent α-synuclein aggregation. Notably, α-synuclein oligomers reciprocally inhibit GCase activity, establishing a pathogenic feedback loop between lysosomal impairment and proteostatic failure [14]. Beyond lysosomal roles, mitochondrial-localized GCase sustains electron transport chain integrity and bioenergetic efficiency [14]. GCase mutations that disrupt their normal function increase oxidative stress in the mitochondria (the region where dopamine is oxidized). In turn, the accumulation of oxidized dopamine adducts further implies GCase activity, creating a second cycle of GCase dysfunction. Oxidative states triggered by GCase dysfunction can also induce mitochondrial DNA damage, which leads to dopaminergic cell death [14]. Astrocytes are an important part of the central nervous system. These cells perform axonal homeostasis and synaptic functions. Glial fibrillary acidic protein (GFAP) is considered to be a marker of astroglia activation. When the central nervous system is damaged, astrocytes release GFAP into the peripheral blood. Some studies have shown that plasma GFAP levels are higher in PD patients compared to healthy controls and are associated with cognitive impairment in PD patients [16]. Axonal integrity is reflected by a neurofilament light chain (NFL), a cytoskeletal component released into biofluids during neurodegeneration. α-Synuclein-driven Lewy pathology disrupts axonal transport, inducing inflammatory NFL leakage. Serum/plasma NFL quantification provides dynamic insights into neuroaxonal injury, complementing clinical and imaging biomarkers for disease staging [18]. DJ-1, also known as Parkinson’s disease protein 7 (Park7), is a multifunctional protein that regulates oxidative stress and mitochondrial function [20]. Under physiological conditions, DJ-1 primarily exists as a homodimer, a flexible and reversible structure that enables its interaction with various partner proteins to perform diverse cellular functions [19]. The dimerization process relies on specific amino acid residues, stabilizing the protein and enabling it to perform its neuroprotective functions. Disruptions in dimerization compromise DJ-1′s stability, increase vulnerability to oxidative stress, and promote protein aggregation [19].

## 3. Parkinson’s Disease Biosensors

A biosensor is a tool that combines a biological element (such as enzymes, antibodies, or DNA) with a transducer to detect and measure specific biological or chemical analytes [23]. Clark et al. proposed a prototype glucose biosensor in 1962 that indirectly measures the amount of glucose in the solution [24,25]. The galvanic glucose biosensor prepared by ferro-electron acceptor in the enzyme REDOX process reported by Cass et al. marks the birth of the second generation of culture medium enzyme biosensors [25,26]. The third generation of biosensors incorporates nanomaterials to amplify signal response and expand the range of application scenarios [25].

The nanomaterials possess abundant active sites, high surface area, and exceptional physical/chemical/electronic properties [8]. These attributes facilitate the immobilization or recognition of biomarkers and confer ultrahigh sensitivity to analytical performance [8]. Importantly, this provides significant advantages for integrating/assembling specialized nanomaterial components to establish biosensors with enhanced analytical performance and commercial viability. In addition, the biosensing platform has been invigorated by the introduction of a diverse range of novel nanomaterials, including graphene, carbon nanotubes, metal nanoparticles, composite nanomaterials, single-atom nanomaterials, and others. Extensive research has been conducted on metal nanomaterials, specifically gold and silver nanoparticles, due to their plasmonic properties. These unique properties enable the amplification of optical signals, resulting in enhanced sensitivity in optical biosensors. Furthermore, metal nanomaterials can be functionalized with biomolecules, such as antibodies or enzymes, to specifically target analytes and thereby enhance selectivity. The synergistic effects of the combined nanomaterials can lead to enhanced properties, such as improved catalytic activity or increased stability. Graphene, a two-dimensional carbon nanomaterial, has emerged as a promising candidate for biosensor applications [27]. Its high electrical conductivity, excellent mechanical strength, and large surface area make it an ideal material for the development of electrochemical biosensors [27]. The integration of nanomaterials into transistor-based biosensors has also been explored. The structure and electrical characteristics of the channel material exert a significant influence on the detection performance of the transistor [23]. Incorporating nanostructures, such as graphene, into the channel material can further enhance the detection performance.

### 3.1. Optical Biosensors in PD Detection

Optical biosensors have been widely utilized in the field of biosensing. It is a type of sensor that operates based on transmitting and collecting light signals [28]. Transducer elements, such as photodiodes, phototransistors, and photoconductive devices, are essential to the conversion of light-based signals in optical biosensors into quantifiable outputs [29]. These sensing elements absorb incident light and generate a corresponding charge or current to detect and analyze biomolecules or compounds. Parkinson’s disease has become a common concern. Its detection, which often relies on the identification of specific biomarkers, has become increasingly important, and optical biosensors have been at the forefront of these diagnostic advances [29].

#### 3.1.1. Dopamine Detection

**Figure 2:**
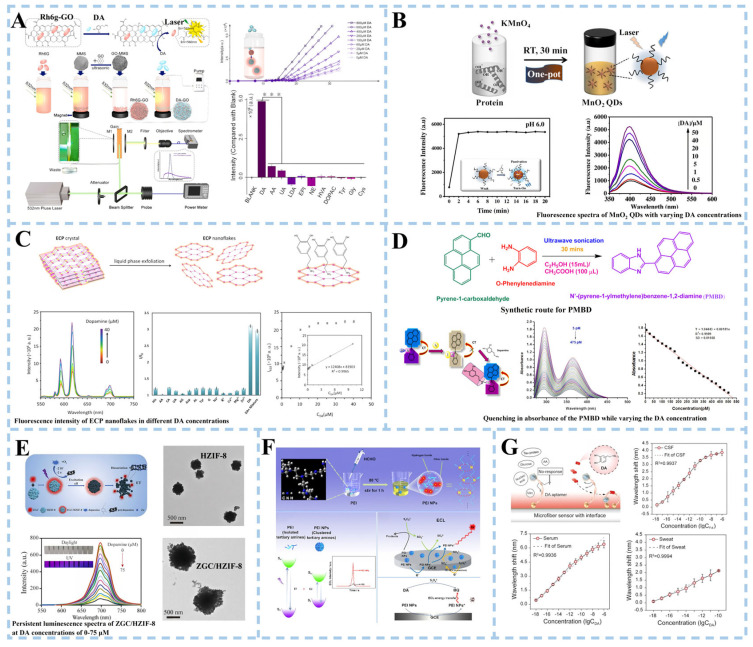
Schematic illustration of an optical biosensor for dopamine detection. (**A**) An optofluidic laser biosensor based on a competitive adsorption mechanism (***: The statistical sig-nificance level *p* < 0.001). Reproduced with permission: Copyright 2022, Elsevier [30]. (**B**) A biosensor based on MnO_2_ quantum dot nanozymes. Reproduced with permission: Copyright 2022, American Chemical Society [32]. (**C**) Dopamine sensing based on ultrathin fluorescent metal−organic nanosheets. Reproduced with permission: Copyright 2020, American Chemical Society [31]. (**D**) Colorimetric sensing using N′-(pyrene-1-ylmethylene)benzene-1,2-diamine/triiodide ions conjugate in human serum. Reproduced with permission: Copyright 2023, Elsevier [33]. (**E**) Optical biosensor based on porous ZIF-8 nanohybrids. Reproduced with permission: Copyright 2022, Elsevier [34]. (**F**) Electrochemical luminescence sensor based on ethyleneimine nanoparticles. Reproduced with permission: Copyright 2022, American Chemical Society [35]. (**G**) Plasma sensor based on optical fiber. Reproduced with permission: Copyright 2023, Wiley-VCH [36]. (MMS: magnetic microspheres, MnO_2_ QDs: Manganese dioxide quantum dots, ECP: [Eu(pzdc)(Hpzdc)(H2O)]_n_, ZGC: Cr^3+^ doped zinc gallate, HZIF-8: zeolite imidazole framework-8, PEI: polyethyleneimine, HCHO: formaldehyde).

Biosensors are highly suitable for the efficient identification of biomarkers associated with Parkinson’s disease in bodily fluids such as cerebrospinal fluid (CSF) and blood. Li et al. [30] proposed a biosensor based on a light flow laser to detect the PD biomarker DA (Figure 2A). Using the competitive adsorption mechanism of Rhodamine 6G (R6G) and DA on the surface of graphene oxide, graphene oxide was combined with magnetic microspheres (GO-MMS) to fluorescence quench Rhodamine R6G, and then, DA solutions of different concentrations were added. The intermolecular interaction of the R6G-GO complex is weaker than DA-GO. With the increase in concentration of DA solution and the increase in free R6G in mixed solution, the relative slope efficiency of the optical flow laser increases, and the laser threshold decreases. At the same time, the Fabry–Perot cavity was also introduced as an optical amplifier to amplify the differences between similar substances, thus achieving selective DA detection. Ideally, the detection limit of DA by an optical flow laser sensor was 0.3 μM. The optofluidic laser sensor showed a way for DA detection in the microcomplex environment. The LOD of 0.3 μM can only facilitate the detection of high concentrations and cannot cover the entire concentration range for healthy individuals. The fluorescence detection method is an effective DA detection method with high selectivity, high sensitivity, fast response, high cost efficiency, and convenient operation [31]. Zou et al. [32] used casein as a reducing agent and stabilizer to synthesize manganese dioxide quantum dot (QD) nanozymes with both fluorescence properties and enzyme simulation activity for DA detection (Figure 2B). The QD nanozymes could catalyze the oxidation of DA to DA quinone (DQ) within 2 min, and the interaction between DQ and MnO_2_ quantum dots played an important role in turning on the fluorescence of QDs. The fluorescence intensity of MnO_2_ quantum dots increased with the increase in DA concentration at PH 6.0. Subsequently, DA in human serum samples was detected and a good recovery rate was obtained, indicating the potential practicability of the sensor based on manganese dioxide QD nanozymes in practical applications. Metal–organic materials (MOMs) have received extensive attention in sensing applications due to the interaction between organic bridging ligands and metal centers and some host–guest supramolecular interactions that enable MOMs to emit or induce fluorescence. Moghzi et al. [31] reported an ultra-thin honeycombed fluorescent europium metal–organic nanomaterial [Eu(pzdc)(Hpzdc)(H_2_O)]_n_ (ECP) for the detection of DA levels (Figure 2C). The results showed that ECP nanosheets had high selective adsorption of DA, and the adsorption molecules of DA could protect ECP nanosheets from the quenching effect of OH oscillators. ECP nanosheets had rapid response and high selectivity for DA detection of clinical samples. They also had a good linear relationship in the range of 0.1~10 μM, and the detection limit was 21 nM.

In addition to fluorescent biosensors, colorimetric biosensors, photoluminescent biosensors, electroluminescent biosensors, and plasma biosensors are also used for DA detection. For example, Servarayan et al. [33] developed a colorimetric sensor using the Schiff base ligand N’-(pyrene-1-ylmethylene)benzene-1,2-diamine along with Triiodide ions (PMBD/triiodide) as a chromophore (Figure 2D). After the addition of dopamine, the PMBD/triiodide solution changed from a slight yellow color to a strong pink color. In the range of 5~475 pM, DA can be selectively detected, exhibiting a limit of detection of 0.02 pM. In the detection of DA in the biological matrix, fluorescence analysis will inevitably be interfered with by spontaneous fluorescence or light scattering due to the presence of impurities such as proteins and other chromophores. Persistent luminescence nanoparticles (PLNPs) are introduced to alleviate this problem. Some researchers proposed constructing a DA detection platform by combining ultra-small Cr^3+^ doped zinc gallate (ZnGa_2_O_4_:Cr^3+^, ZGC) sustained luminescence nanoparticles with layered porous zeolite imidazole framework-8 (HZIF-8) [34] (Figure 2E). The abundant stratified pores improve the stability of ZGC/HZIF-8 composites in the HZIF-8 matrix. With the increase in DA concentration, the red fluorescence of ZGC/HZIF-8 decreased, and the sustained luminescence intensity decreased gradually. The reason was the transfer of photoexcited electrons from ZGC to the oxidized PDA (the oxide of DA), resulting in the luminescence quenching of the ZGC/HZIF-8 composite. The sensor was validated for dopamine (DA) quantification in human serum samples from healthy individuals. When DA was spiked into a 100-fold diluted serum matrix, the method demonstrated a recovery rate of 98.2–104.7%. These results confirm the sensor’s reliability for precise quantitative detection of DA in complex biological matrices. Electrochemiluminescence (ECL) is a combination of chemiluminescence and electrochemical technology. The adhesion of the luminescent body to the electrode surface and the interaction between the quencher substance and the luminescent body play an important role in the detection of ultra low-level biomolecules [37]. In addition, efficient and low-cost luminescent groups have been the focus of research in electrochemical luminescence sensors. Zhang et al. [35] synthesized polyethyleneimine nanoparticles (PEI NPs) with cluster luminescence properties using a one-step hydrothermal method, and constructed a ternary electrochemical luminescence (ECL) sensor (Figure 2F). The presence of persulfate oxidizes dopamine to benzoquinone (BQ) and the quenching signal were transferred by ECL energy from PEI NPs to BQ. ECL intensity decreased with the increase in dopamine concentration. Plasmon resonance can effectively improve the sensitivity of fiber-optic biosensors. Therefore, Huang et al. [36] developed a fiber-optic biosensor based on the double-amplified nanointerface of plasma coupling and conformational aptamer transformation to achieve the detection of DA at the single-molecule level (Figure 2G). The detection limits of DA molecules in CSF, serum, and artificial sweat were 1.30 aM, 1.53 aM, and 0.54 aM, respectively.

#### 3.1.2. α-Synuclein Detection

**Figure 3:**
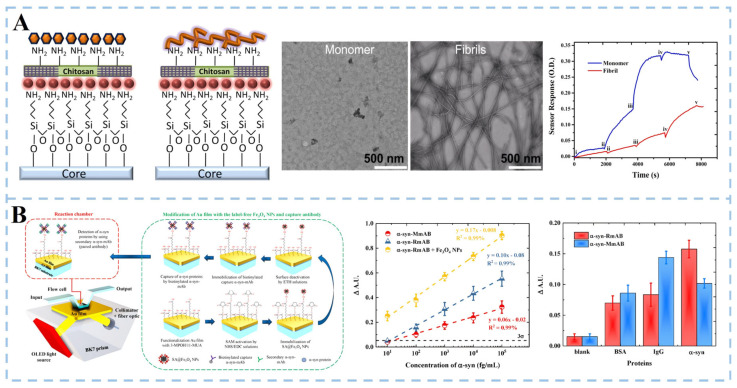
Schematic illustration of optical biosensor for α-synuclein detection. (**A**) LSPR sensor based on chitosan film-coated nanogold array (i: 70 nM, ii: 175 nM, iii: 350 nM, iv: 700 nM, v: washing with buffer). Reproduced with permission: Copyright 2017, Elsevier [38]. (**B**) SPR biosensor utilizing paired antibody and label-free Fe_3_O_4_ nanoparticles. Reproduced with permission: Copyright 2021, Multidisciplinary Digital Publishing Institute [39].

The aggregation of soluble α-synuclein (α-syn) is another key pathological marker of PD. Local surface plasmon resonance (LSPR) and plasmon resonance (SPR) techniques amplify the signal by exciting the surface plasma on the metal surface. In this way, Khatri et al. [38] developed an LSPR biosensor (Figure 3A). The amino-silanization sensor probe was immersed in a colloidal solution of gold nanoparticles (GNP) to form a GNP monolayer array, which was then functionalized with a chitosan film to form a sensing matrix. The sensor can independently detect monomers and fibrils at a concentration of 70 nM and distinguish them specifically. Mandala et al. [39] developed an SPR biosensor based on unlabeled iron oxide nanoparticles (Fe_3_O_4_ NPs) and paired antibodies (Figure 3B). The surface of the sensor chip gold film was modified with a mixed self-assembled monolayer (SAM) composed of 3-mercapto-1-propanol (3-MPOH) and 11-mercaptoundecanoid (11-MUA). Fe_3_O_4_ NPs labeled with streptavidin (SA) were functionalized. Finally, biotinylated monoclonal antibodies (α-syn-RmAb or α-syn-MmAb) are fixed to SA@Fe_3_O_4_ NPs by avidin–biotin interaction. The results show that the sensitivity of the SPR platform is significantly increased by 1.7 times when high-density Fe_3_O_4_ nanoparticles are deposited on the surface of the Au sensor. Compared with murine monoclonal antibodies, rabbit monoclonal antibodies have stronger binding to α-syn molecules (α-syn-RmAb) and higher selectivity for antigen detection. The SPR sensor uses the paired α-syn-RmAb format to detect α-syn in diluted serum samples accurately. It paves the way for the future development of early diagnosis of PD.

Furthermore, many researchers have used modified α-synuclein (such as nitration and phosphorylation) as a marker of PD for biosensor detection and have obtained good detection sensitivity. Hu et al. [40] proposed a sandwich structure strategy for nitro-α-synuclein detection. Nitro-syn was obtained by modifying α-Syn with 3-Morpholinosydnonimine (SIN-1). Nitro-syn exists on the Tyr 39 residue (nT39 α-Syn) and was an essential component of aggregation α-Syn. Metalloporphyrin (FeP) was self-assembled with supramolecular-modified gold nanoparticles (pSC4-AuNPs) to obtain nanozyme (FpA), and the α-Syn antibody was combined to form Ab-FpA. The gold surface of the sensor chip was modified with 11-MUA, activated by 1-(3-dimethylaminopropyl)-3-ethylcarbodiimide hydrochloride (EDC) and n-hydroxysulfosuccinimide (NHS). Finally, the nT39α-Syn antibody (nT39α-Syn Ab) was added, and a sandwich structure was constructed with nT39α-Syn and Ab-FpA. The sensor used the LSPR effect and the mass effect caused by FpA catalytic deposition to realize signal amplification of nT39 α-Syn detection. The platform demonstrated a linear detection range of 7–240 ng/mL and a limit of detection (LOD) of 1.78 ng/mL. However, the sensitivity of this approach was constrained by the catalytic efficiency of FpA. Validation using serum samples from healthy volunteers and Parkinson’s disease (PD) patients revealed that targeting nT39 α-Syn significantly improved diagnostic sensitivity for PD. These results show the clinical feasibility of the method, highlighting its potential for integration into standardized PD diagnostic workflows. 90% of the α-synuclein aggregated in Lewy’s bodies is phosphorylated. Phosphorylated α-synuclein (p-αS) can interact with cytoskeletal proteins to block vesicular transport, enhance the aggregation of α-synuclein in vivo and in vitro, induce the formation of toxic aggregates, and accelerate the progress of PD. Yin et al. [41] used a dual-channel SPR method to evaluate α-synuclein phosphorylation levels quantitatively. Similarly, 11-MUA was used to modify gold chips. EDC and NHS were injected into the reference channel and analysis channel, respectively. The total α-synuclein (t-αS) was quantified by one anti-αS antibody, which effectively eliminated the uncertainty caused by changes in the interface structure and microenvironment, and improved the detection accuracy of t-αS. The specific recognition of p-αS by titanium-phosphate nanoparticles (Ti^4+^@TiP) improved the detection sensitivity. The results showed that the dynamic range of t-αS was 1.0~20.0 pg mL^−1^, and the dynamic range of p-αS was 0.1~10.0 pg mL^−1^. The results also proved that it was feasible to determine the concentration of αS and p-αS in clinical cerebrospinal fluid samples. In addition, detailed information about different optical sensing for DA and α-syn detection is described in Table 1.

#### 3.1.3. Other Biomarker Detection

In addition to dopamine and α-syn, optical sensors have also been used to detect other biomarkers of Parkinson’s disease. For example, Xiao et al. [42] engineered a gold nanostar interface optical fiber sensor for the detection of Glial fibrillary acidic protein (GFAP). The sensor demonstrated a limit of detection of 0.09 aM in phosphate-buffered saline (PBS), achieving single-molecule resolution for GFAP quantification. In parallel, Varghese et al. [43] developed a fluorescence sensor to detect GFAP. Employing a molybdenum disulfide nanosheet (MoS_2_ NS) as a quencher upon GFAP antibody-conjugated bovine serum albumin-capped fluorescent gold nanoclusters (MoS_2_@Ab@AuNCs). The system demonstrated a linear detection range of 31.15–447.76 pg/mL and a limit of detection (LOD) of 1.30 pg/mL for GFAP. While GFAP serves primarily as an auxiliary PD biomarker with prognostic value for disease staging, its clinical utility requires multiplexed analysis with core pathological indicators such as α-synuclein to enhance diagnostic specificity.

**Table 1:** Different optical sensing schemes for Parkinson’s disease biomarker detection.

Target	Method	Nanoparticles	Response Time	Linear Range	LOD	Selectivity	Stability	Ref
DA	Fluorescence	Si NPs	-	1–10 μM, 10–50 μM	0.22 μM	AA, UA, Tyr, Glu, NE, EP, Gly	15 days	[44]
	Fluorescence	ECP	5 min	0.1–10 μM	21 nM	AA, UA, His, Tyr, Glu, Arg, Asp, AL, Na^+^, K^+^	5 times	[31]
	Fluorescence	GO	-	3–1680 nM	0.031 nM	Gly, Cys, Glu, Lac	5 times	[45]
	LSPR	Cu_2−x_S@GO, Au@MoS_2_	Seconds to minutes	-	0.45 aM	UA, AA, Glu, NA, Tau	-	[36]
	Colorimetric	-	2 min	5–475 pM	0.02 pM	UA, urea, AA, Glu, creatine, Na^+^, K^+^	5 times	[33]
	ECL	CDs	-	0.01–100 μM	62 nM	AA, UA	14 times	[46]
	Colorimetric	CoFe_2_O_4_	135 s	5–80 μM	0.233 μM	Gly, GSH, Lys, AA, UA, Cu^2+^, Fe^2+^, Na^+^, K^+^	-	[47]
	ECL	PEI NPs	-	10 nM–10 μM	43 nM	AA, His, Glu, Cys	18 times	[35]
	ECL	Ir@MXene-PVA	-	0.01–100 μM	2 nM	AA, HVA	8 times	[48]
	SERS	Au NPs/GNS/EPR	3 s	5–90 μM	3.3 μM	-	-	[49]
	Colorimetric	Bi_2_Fe_4_O_9_ NPs	-	0.15–50 μM	51 nM	Cys, Glu, AA, GSH, Lys, Ala, Arg	4 times	[50]
	LSPR	AuNRs@PANI	-	-	7.11 μM	-	-	[51]
	SERS	Ag NPs	-	1 fM–10 pM	10 aM	-	7 days	[52]
	Fluorescence	UCNPs	-	0–200 μM	83.6 nM	UA, AA, Glu, Gly, Na^+^, K^+^, SAM	-	[53]
	Fluorescence	N,Cl-CDs	-	10–90 μM	0.1212 μM	-	-	[54]
	Colorimetric	Co-MOFs/MoS_2_	-	1–10 μM	0.83 μM	AA, UA, Glu	12 times	[55]
α-syn	Fluorescence	CDs	5 min	0.5–10 μM	59 nM	Cys, Arg, His, Na^+^	-	[56]
	SPR	Au	-	50–250 nM	5.1 nM	-	5 times	[57]
	Liquid crystal	-	-	50–400 nM	50 nM	Lysozyme, α-lac, PK, Str	-	[58]
	SPR	FpA	-	7–240 ng/mL	1.78 ng/mL	AA, Glu, BSA,	-	[40]
	SPR	Ti^4+^@TiP		1–20 pg/mL 0.1–10 pg/mL (t-αS, p-αS)	0.07 pg/mL, 0.032 pg/mL (t-αS, p-αS)	Aβ_40_, Aβ_42_	≥1 month	[41]
	Colorimetric, SPR	Au NPs		20 nM–3 μM, 0.1 nM–0.5 μM	10 nM, 8 pM,	BSA, lysozyme, LgG, Thr	-	[59]
	Fluorescence	Eu-MOF@Au	-	0.0005–1 ng/mL	0.04 pg/mL	Glu, BSA, lipid, DNase, DNase	-	[60]
GFAP	Optical microfiber	Au NSs	-	1 aM–0.1 nM	0.09 aM	Na+, Glu, S100B, IL-6, AFP	-	[42]
	ECL	CMCS@ATT-AgAu BMNCs	-	100 ag/mL–160 pg/mL	73 ag/mL	LgG, CEA, AFP	10 times	[61]
	Fluorescence	MoS_2_@Ab@AuNCs	10 min	31.15–447.76 pg/mL	1.30 pg/mL	Glu, creatine, BSA, UA, Cys, Arg, Na^+^, K^+^	5 days	[43]
	Fluorescence	CDs	-	10 pg/mL–10 ng/mL	2.2 pg/mL	BSA, CPR	7 days	[62]

ECP: [Eu(pzdc)(Hpzdc)(H_2_O)]_n_ (H_2_pzdc: 2,3-pyrazine dicarboxylic acid), PPy/GQDs: polypyrrole/graphene quantum dots, LSPR: localized-surface plasmon resonance, ZGC: ZnGa_2_O_4_:Cr^3+^, HZIF-8: hierarchical porous zeolite imidazole framework-8, ECL: electrochemiluminescence, PEI NPs: polyethyleneimine nanoparticles, Ir: iridium bis(1,2-dipheny1–1H-benzimidazole)(acetylacetonate) [Ir(pbi)2(acac)], PVA: polyvinyl alcohol, GNS: graphene nanosheets, EPR: epoxy resin, AuNRs@PANI: polyaniline-coated gold nanorods nanocomposites, UCNPs: upconversion nanoparticles, CDs: carbon dots, FpA: iron porphyrin-mediated supramolecular-modified gold nanoparticles, Ti^4+^@TiP: titanium phosphate nanoparticles, t-αs: total alpha-synuclein, p-αs: phosphorylated alpha-synuclein, Eu-MOF: europium-based metal–organic framework, Au NSs: gold nanostar, CMCS@ATT-AgAu BMNCs: carboxymethyl chitosan (CMCS)@6-aza-2-thiothymine (ATT) templated AgAu bimetallic nanoclusters, CDs: carbon dots, AA: ascorbic acid, UA: uric acid, Tyr: tyrosine, Cys: cysteine, NE: norepinephrine, EP: epinephrine, HVA: homovanillic acid, Glu: glucose, His: histidine, Arg: arginine, Asp: aspartic acid, AL: alanine, Gly: glycine, Lac: lactic acid, NA: nucleic acid, Tau: tubulin-associated protein, GSH: glutathione, BSA: bovine serum protein, Lys: lysine, Ala: alanine, SAM: S-adenosylmetionine, LgG: immunoglobulin, α-lac: α-lactalbumin, PK: proteinase K, Str: streptavidin, Aβ_42_ and Aβ_40_: Alzheimer’s disease-associated proteins, Thr: thrombin, DNase: deoxyribonuclease, S100B and IL-6: non-specific proteins, CEA: carcinoembryonic antigen, AFP: alpha fetoprotein, CPR: C-reactive protein.

### 3.2. Electrochemical Biosensors in PD Detection

Electrochemistry is a powerful tool in biosensing, integrating biological recognition events, and converting specific analyte information into measurable electrochemical signals [63]. The transduction component of an electrochemical biosensor is typically an electrochemical cell with the primary element being a working electrode [64]. In a potentiostatic system, it is common to use a three-electrode format consisting of working, auxiliary, and reference electrodes. For conductometry and electrochemical impedance spectroscopy (EIS), a two-electrode format with working and auxiliary electrodes is often employed [64]. Electrodes are primarily fabricated using glassy carbon, gold, platinum, or indium tin oxide due to their high chemical inertness and wide potential window [63]. Commonly, electrodes based on modifications such as carbon-based nanomaterials or nanocomposites are used to improve the sensitivity and selectivity of the assay.

#### 3.2.1. Dopamine Detection

**Figure 4:**
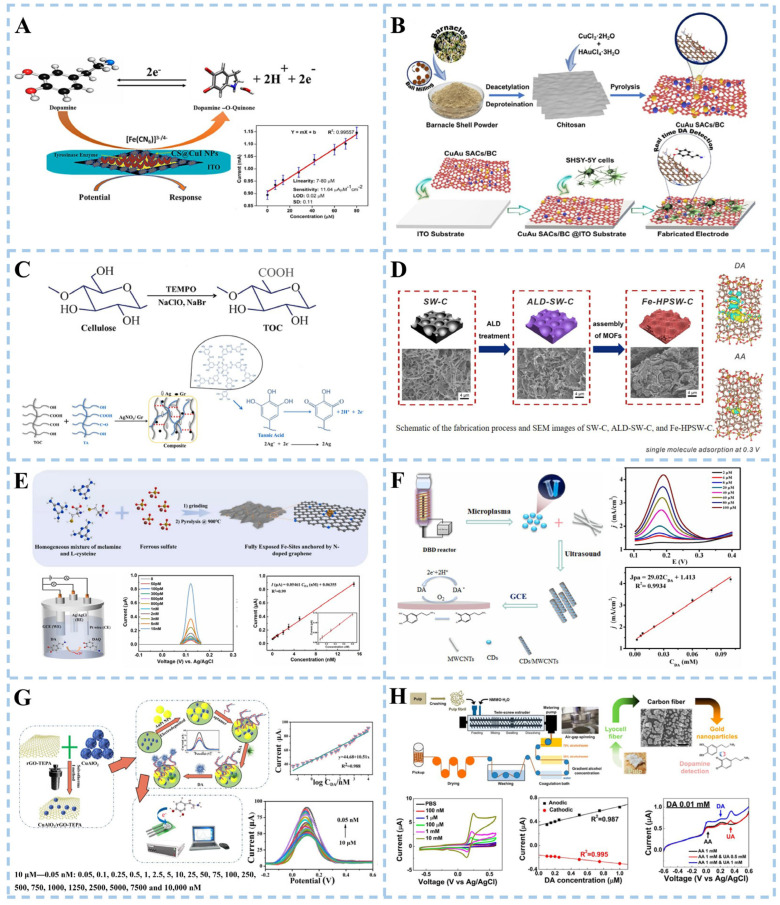
Schematic illustration of electrochemical biosensor for dopamine detection. (**A**) Electrochemical biosensing of chitosan-stabilized copper iodide nanoparticles. Reproduced with permission: Copyright 2023, Elsevier [65]. (**B**) Electrochemical sensing based on bimetallic monatomic catalysts. Reproduced with permission: Copyright 2023, Elsevier [66]. (**C**) Electrochemical sensing based on TEMPO-oxidized cellulose composites. Reproduced with permission: Copyright 2023, Elsevier [67]. (**D**) Electrochemical sensing based on metal–organic framework. Reproduced with permission: Copyright 2023, American Chemical Society [69]. (**E**) Electrochemical sensing based on oligomeric graphene. Reproduced with permission: Copyright 2022, Elsevier [70]. (**F**) Electrochemical sensing based on carbon nanocomposites. Reproduced with permission: Copyright 2023, Elsevier [71]. (**G**) Electrochemical sensing based on CuAlO_2_/rGO-TEPA@AuPt nanocomposites. Reproduced with permission: Copyright 2022, Elsevier [72]. (**H**) Electrochemical sensing based on gold nanoparticle/carbon fiber composites. Reproduced with permission: Copyright 2022, American Chemical Society [74]. (CS@CuI NPs: chitosan-stabilized copper iodide nanoparticles, CuAu SACs/BC: copper and gold atoms fixed on an electrode made of bioinspired chitosan-extracted carbon, TEMPO: cellulose modified by 2,2,6,6 tetramethylpiperidine-1-oxyl, TOC: TEMPO-oxidized cellulose, ALD: atomic layer deposition, Fe-HPSW-C: Fe-doped layered porous algal carbon, CDs: carbon quantum dots, CuAlO_2_/rGO-TEPA: copper aluminate/reduced graphene oxide-tetraethylene pentaamine, NMMO: N-oxide aqueous solution).

Conducting and semiconducting ceramics, such as indium tin oxide (ITO), have been commonly used for dopamine detection. Singh et al. [65] modified chitosan-stabilized copper iodide nanoparticles (CS@CuI NPs) onto ITO electrodes and immobilized tyrosinase (Tyr) onto the modified electrodes to prepare Tyr/CS@CuI NPs/ITO bioelectrodes (Figure 4A). Copper iodide nanoparticles (CuI NPs) are easy to functionalize and help to fix Tyr. They also provide better REDOX activity, solubility, and enhanced surface reactivity, which plays a good role in electrode manufacturing. The biosensor based on the electrode has good performance in detecting dopamine. The electrochemical sensor exhibited a sensitivity of 11.64 μA μM^−1^ cm^−2^, with a detection limit of 0.02 μM. Chellasamy et al. [66] modified the electrode with bimetallic single-atom catalysts (SACs) (Figure 4B). Singular copper and gold atoms were fixed on an electrode made of bioinspired chitosan-extracted carbon (CuAu SACs/BC). Chitosan as a carrier avoided the aggregation of single atoms. The sensing of dopamine is primarily attributed to individual Cu atoms, while the presence of individual Au atoms further enhances signal detection. The synergistic effect of the two enabled the CuAu SACs/BC electrode to detect dopamine at the nanomolar level in the plasma of elderly men and women.

The glassy carbon electrode made of composite material composed of glass beads and epoxy resin is also one of the electrodes commonly used in electrochemical biosensors. Al Kiey et al. [67] conducted a direct electrochemical approach to explore the detection of dopamine. Cellulose was first modified by 2,2,6,6 tetramethylpiperidine-1-oxyl (TEMPO) to generate nanocellulose with many carboxyl groups. Ag^+^ was reduced to Ag^0^ and promoted the formation of TEMPO-oxidized cellulose/silver nanoparticles/graphite (TOC/AgNPs/Gr) nanocomposite in the presence of tannic acid as a reducing agent and crosslinker (Figure 4C). The TOC/AgNPs/Gr nanocomposite sensor exhibited a linear response range of 0.05–250 μM and a limit of detection of 0.005 μM, as determined by differential pulse voltammetry (DPV). The platform demonstrated a sensitivity of 0.963 μA·μM^−1^·cm^−2^, highlighting its robust electrochemical performance. To validate clinical utility, the sensor was tested in human urine and serum samples, achieving recovery rates of 94.20–98.00% (urine) and 94.16–97.14% (serum) for dopamine quantification. These results confirm the method’s reliability for dopamine detection in complex biological matrices. Sun et al. [68] developed an electrochemical biosensor with high sensitivity for the detection of DA. The hydrothermal process was employed to synthesize flower-like MoS_2_, followed by recrystallization of the nickel complex and MoS_2_ homogeneous solution at 80 °C. Finally, pyrolysis in a tube furnace obtained the flower-like MoS_2_ modified by an Ni single atom. The single Ni atom was located directly above the Mo atom, significantly enhancing the electrochemical signal and catalytic activity toward DA. The detection limit of DA achieved by the electrochemical biosensor reached 1 pM. Density functional theory (DFT) results further confirmed that the Ni atom exhibited stronger sensitivity and selectivity towards DA adsorption and oxidation. Metal–organic framework (MOF) films are also commonly used for electrode modification. To improve the fabrication performance of the MOF film-based electrodes. Ke et al. [69] prepared Fe-doped MOF (MIL-53 (Fe)) with carbonized seaweed as a substrate and formed Fe-doped layered porous algal carbon (Fe-HPSW-C) sensor electrodes with the aid of atomic layer deposition (ALD) ZnO nanofilms (Figure 4D). The algae carbon substrate promoted electron transfer and reduced the disadvantage of low conductivity of MOFs. The ZnO nanomembrane was deposited by ALD to facilitate the subsequent preparation of MOF films with strong adhesion. The MIL53 (Fe) framework exhibited a high degree of active site exposure and a multitude of catalytically active ions, thereby significantly enhancing sensitivity. The obtained sensor demonstrated excellent performance and effectively distinguished between DA and ascorbic acid at an optimized potential of 0.3 V. Sun et al. [70] synthesized the nitrogen–carbon-supported Fe-based subnanometric catalyst with fully exposed Fe sites (Fe/N-GR) by calcinating a mixture of melamine, L-cysteine, and FeSO_4_ at 900 °C in a flowing N_2_ atmosphere to construct an electrochemical biosensor for DA detection (Figure 4E). The fabricated Fe/N-GR biosensor achieves enzyme-like activity and a fast response toward DA oxidation. The biosensor demonstrated a low detection limit of 27 pM and exhibited a wide linear dynamic range spanning from 50 pM to 15 nM. The remarkable enzyme-like specificity of the Fe/N-GR catalyst towards DA can be ascribed to the fully exposed Fe-sites and the active Fe-N coordination structure, which contribute to the selectivity of Fe/N-GR/GCE. The biosensor obtained a good recovery rate for detecting DA in actual human serum samples. Zhou et al. [71] synthesized carbon quantum dots (CDs) using glycerol as a carbon source through liquid dielectric barrier discharge (DBD) microplasma technology (Figure 4F). A composite electrode of carbon nanomaterial was constructed by combining CDs with highly conductive multi-wall carbon nanotubes (MWCNTs) for DA detection. The electrode exhibited a low detection limit of 11.08 nM and has been successfully applied for detecting DA in human serum.

A thick-film carbon electrode (screen printing electrode) is prepared by printing technology and is widely used in electrochemical sensors and energy storage devices. The effective detection of dopamine was obtained by modifying the electrode. Wei et al. [72] proposed an electrochemical sensor based on DNA aptamers (Figure 4G). A hydrothermal reaction prepared a copper aluminate (CuAlO_2_)/reduced graphene oxide-tetraethylene pentamine (rGO-TEPA). Subsequently, gold and platinum nanoparticles (AuPt NPs) were deposited on the surface of the modified electrode using excess oxygen, resulting in a strong promoting effect between CuAlO_2_ and rGO-TEPA, which significantly enhances electron transfer on the surface of the immunosensor. Moreover, bimetallic AuPt NPs provided additional attachment sites for the aptamer, thereby improving biosensor sensitivity. The developed immunosensor demonstrated real-time monitoring capabilities within a concentration range of 0.05 nM to 10 μM, with a detection limit of 0.017 nM at S/N = 3. However, its current limitation lies in its specificity towards dopamine detection, which restricts its clinical applicability. Rana et al. [73] developed a carbon-based nanomaterial electrochemical biosensor from the extract of Parthenium hysterophorus (one of the top seven dangerous weeds) leaves used for the detection of DA. In the carbon nanomaterial synthesis process, the sulfuric acid-treated extract was subjected to hydrothermal treatment in a muffle furnace. The resulting product was washed, dried, and then carbonized in a tube furnace. Finally, the stable carbon nanomaterial remained after high-temperature treatment. This method eradicated Parthenium hysterophorus and showed good electrochemical and sensing properties for DA.

The combination of carbon fiber and metal nanoparticles can be used directly as electrode materials. Lim et al. [74] proposed an environmentally friendly and sustainable approach for synthesizing carbon fiber/gold nanoparticles to enable the detection of DA (Figure 4H). The process involved introducing pulverized pulp and evaporated 4-methylmorpholine N-oxide aqueous solution (NMMO) into a double-screw extruder, followed by extrusion in ambient air and subsequent passage through a coagulation pool, solidification pool, washing zone, and drying zone to obtain lyocell fiber (LF). Subsequently, high-performance LF was subjected to thermal treatments such as tension stabilization and carbonization to transform it into cellulose-based carbon nanofibers (CF). This well-designed structure exhibited remarkable sensitivity toward DA with a value of 0.320 μA/μM, along with an appropriate limit of detection (LOD) of 25 nM. Furthermore, the structure demonstrated excellent selectivity under brain fluid conditions.

#### 3.2.2. α-Synuclein Detection

α-synuclein (α-syn) undergoes misfolding, resulting in the formation of oligomers that subsequently aggregate into α-syn fibers, posing a potential threat to neuronal integrity and contributing to the onset of Parkinson’s disease. Therefore, precise monitoring of α-syn, a key biomarker for PD, is imperative for early detection. Electrochemical biosensors have gained significant traction in α-syn detection owing to their exceptional sensitivity, rapid response time, and user-friendly operation.

**Figure 5:**
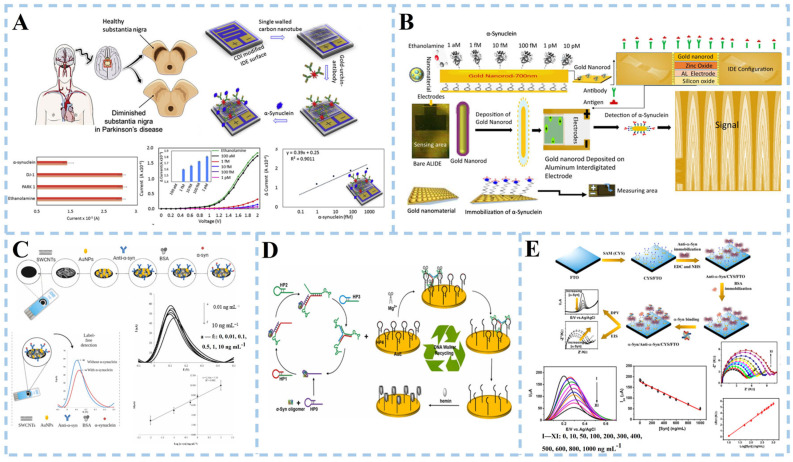
Schematic illustration of electrochemical biosensor for α-synuclein detection. (**A**) Sensor based on gold-nanourchin–antibody modified. Reproduced with permission: Copyright 2020, Elsevier [75]. (**B**) Sensor based on ZnO-gold nanorods. Reproduced with permission: Copyright 2021, Elsevier [76]. (**C**) Sensor based on AuNPs/SWCNTs. Reproduced with permission: Copyright 2023, Elsevier [77]. (**D**) Sensor based on the MNAzyme-driven tripedal DNA walker strategy. Reproduced with permission: Copyright 2023, Elsevier [78]. (**E**) Sensor based on self-assembled monolayer modifications. Reproduced with permission: Copyright 2020, Multidisciplinary Digital Publishing Institute [79].

Interdigitated electrode (IDE) sensors are based on electrochemical reactions and capacitive effects. The target analyte reacts with the sensor electrode to generate an electrical signal proportional to the concentration of the target analyte. It is a label-free system, where probes such as RNA, DNA, protein, antibody, and aptamer are used to transduce biomolecular interactions into readable electric signals. Zhang et al. [75] developed a highly sensitive IDE electrochemical biosensor for α-syn detection by modifying the IDE surface (Figure 5A). Single-walled carbon nanotubes (SWCNTs) were deposited onto an IDE modified with Carbonyldiimidazole (CDI), chemically functionalized by gold-urchin conjugated antibodies, and used for the detection of α-syn. The results demonstrated a limit of fibril-formed α-synuclein detection at 1 fM concentration. Furthermore, selectivity experiments involving closely related proteins mutant alpha-synuclein (PARK 1) and oxidative stress (DJ-1) revealed clear discrimination towards α-syn without fouling, while exhibiting higher selectivity and reproducibility. Adam et al. [76] fabricated electrochemical aluminum cross-finger electrodes (ALIDEs) using conventional lithography (Figure 5B). Zinc oxide and gold nanorods nanocomposites were modified on the electrode surface to analyze α-syn at different concentrations. α-syn in aggregated, non-aggregated, and aggregated labeled serum samples were studied.

Screen-printed carbon electrodes (SPCE) are commonly utilized for the detection and quantification of α-syn through surface modification. Electrochemical immunosensors have been developed using screen-printed carbon electrodes. This involved applying an appropriate amount of MWCNTs to SPCE, followed by the deposition of gold nanoparticles on MWCNTs-modified SPCE, and functionalizing the nanostructured surface with monoclonal antibodies [77] (Figure 5C). Modifications of SWCNTs and AuNPs enhance electron transfer at the electrode–electrolyte interface, improving the electrochemical response of SPCE while also increasing its long-term stability. The detection range for α-syn is 0.01~10 ng mL^−1^, with a detection limit of 4.1 pg mL^−1^ and a limit of quantification at 12.6 pg mL^−1^. Accurate recovery rates were achieved after adding standards in fetal bovine serum, demonstrating good specificity and stability within one month.

DNA walkers have been extensively utilized in the detection of α-syn oligomers due to their ability to amplify cascade signals triggered by autonomous motion. However, current research on multi-legged DNA walkers has primarily focused on employing intricate procedures for immobilizing walker DNA onto gold nanoparticles. Gong et al. [78] devised an electrochemical biosensor based on a gold electrode and successfully employed it for the detection of α-syn oligomers (Figure 5D). The researchers employed α-syn oligomers as guides for DNA self-assembly, facilitating the formation of three-legged DNA walkers without requiring the attachment of the DNA walking strand to gold nanoparticles. Mg^+^ acts as a cofactor for DNAzyme, resulting in MNAzyme formation and propelling continuous cutting of HPs (nucleic acid fragments) by the three-legged DNA walker. The walker autonomously traverses functionalized track chains on gold electrodes, while hemoglobin binds with an unlocked G-tetramer (a unique DNA structure) during its movement to form a G-tetramer/hemoglobin complex. Iron ions within hemoglobin undergo reversible REDOX reactions between Fe (III) and Fe (II), with the G-tetramer serving as a medium for electron transfer, significantly enhancing current output. This alteration in current can be detected as an indication of the presence of α-syn oligomers. The electrochemical aptamer sensor demonstrated quantitative detection of α-synuclein (α-syn) oligomers in human serum samples, with performance validated against conventional electrochemiluminescence (ECL) detection. Comparative analysis revealed relative standard deviations (RSD) of 3.31–4.14% for ECL and 3.47–4.45% for the proposed sensor.

Ge and colleagues presented a label-free electrochemical sensor that used a cystamine (CYS) self-assembled monolayer (SAM) to adorn a fluorine-doped tin oxide (FTO) electrode for α-Syn detection [79] (Figure 5E). The adsorption of CYS molecules through the head sulfur groups resulted in the formation of CYS-SAM onto the FTO electrode. The appropriate proportions of EDC and NHS facilitated the coupling between the -NH_2_ groups of CYS and the carboxyl groups (-COOH) of anti-alpha-syn, forming amide bonds. The role of BSA was to mitigate the non-specific binding of α-syn antigens, thereby enhancing the specificity of the assay. A low detection limit of 3.62 and 1.13 ng/mL was achieved in differential pulse voltammetry (DPV) and electrochemical impedance spectra (EIS) measurements, respectively. In addition, details of the different electrochemical biosensors for DA and α-syn detection are shown in Table 2.

#### 3.2.3. Other Biomarker Detection

Beyond DA and α-syn, detecting additional PD-related biomarkers is clinically imperative for comprehensive pathological characterization [80]. Using a novel reduced graphene oxide/polydopamine molecularly imprinted polymer (rGO/PDA-MIP) as the probe layer, Li et al. [81] demonstrated an electrochemical sensing platform for ultra-sensitive detection of GFAP. The sensor exhibited a limit of detection (LOD) of 754.5 ag/mL and demonstrated clinical validity in plasma analysis, with recovery rates of 81.6–108.8% relative to single-molecule array assays, underscoring its utility in neurodegenerative disease monitoring. In parallel, electrochemical strategies for Parkinson’s disease (PD)-associated neurofilament light chain (NfL) detection has advanced. Ozgur et al. [82] developed a biosensor employing glycidyl methacrylate (GMA)-functionalized electrodes covalently immobilized with anti-NfL antibodies. achieving an LOD of 5.21 ng/L. This covalent biomolecular interface enhanced specificity for NfL quantification in complex matrices. Recent efforts have also targeted DJ-1, a redox-sensitive PD biomarker. Kumar et al. [83] prepared molecularly imprinted polypyrrole (MIPPy) for DJ-1 protein recognition on the surface of a screen-printed carbon electrode (SPCE) and prepared a Au NP conjugate impedance electrochemical sensor. The platform achieved a linear dynamic range of 1–500 nM and an LOD of 1 nM, enabling precise quantification of DJ-1 in blood and cerebrospinal fluid (CSF).

**Table 2:** Different electrochemical sensing schemes for Parkinson’s disease biomarker detection.

Target	Technique	Nanoparticles	Electrode	Response Time	Sensitivity	Linear Range	LOD	Selectivity	Stability	Ref
DA	DPV	-	ZTO/ITO	-	11.057 μAμg^−1^ mL cm^−2^	0.1–1 μM	0.013 μM	UA, AA, BSA,	≥4 years	[84]
	DPV	MXene-Au	GCE	-	-	0.1–100 μM	0.04 μM	UA, AA	11 times	[85]
	CA	MnO_2_ NSs	GCE	20 s	-	0.01–20 μM, 20–100 μM	4.1 nM	AA, UA	9 days	[86]
	DPV	Au-S_v_-MoS_2_- CNTs	GCE	-	0.0046 μA nM^−1^	2–900 nM	2 nM	K^+^, Na^+^, Glu, Gly, AA, UA	30 days	[87]
	DPV, Amperometry	TOC/AgNPs/Gr	GCE	-	0.963 μAmΜ^−1^ cm^−2^	0.005–250 μM	0.0005 μM	UA, AA, Glu	-	[67]
	DPV	CuAu SACs	ITO	50 s	17.15 μAμM^−1^ cm^−2^	1 nM–1 μΜ, 1–100 μM	200 pM	AA, UA, Glu, Na^+^, K^+^, EP	10 days	[66]
	CV, DPV	CGC-500	SPCE	-	13.75 μAμM^−1^ cm^−2^, 3.31 μAμM^−1^ cm^−2^	0.1–10 μM	0.6 μM, 0.8 μM	UA, AA, Glu	60 days	[73]
	DPV	SiC/graphene	Si	-	0.86 μAμM^−1^ cm^−2^	0.5–78 μM	0.11 μM	UA, AA, Na^+^, K^+^	-	[88]
	CV, Amperometry	Fe-HPSW-C	GCE	-	2084.58 μA mM^−1^ cm^−2^	1.0–200 μM	0.73 μM	K+, Na+, THAM, Glu, AA	15 h	[69]
	DPV	Fe/N-GR	GCE	2.5 s	0.05461 μA nM^−1^	50 pM–15 nM	27 pM	Lactate, Glu, urea, adenosine, Thy	35 days	[70]
	DPV	CDs/MWCNTs	GCE	-	29,020 μAcm^−2^ mM^−1^	2–100 μM	11.08 nM	AA, UA, Na^+^, K^+^, Cl^−^, NO^3−^	-	[71]
	DPV	Ni-MoS_2_	GCE	-	-	1 pM–1 mM	1 pM	Glu, urea, UA, AA, Na^+^, K^+^	7 days	[68]
	DPV	CuAlO_2_/rGO-TEPA@AuPt	SPCE	Real-time	-	0.05 nM–10 μM	0.017 nM	UA, AA, L-cys, Glu	30 days	[72]
	CV	Carbon fiber/gold	-	-	0.320 ± 0.022 μA μM^−1^	0.1–10 μM	25 nM	UA, AA	-	[74]
	DPV, CA	MPTMS@ Yb_2_O_3_	GE	-	1.88 μA mM^−1^ cm^−2^	1–40 μM	41 nM, 77 nM	Glu, urea, AA	100 times, 5 days	[89]
	CV, DPV	In_1_−N−C	GCE	-	8.24 μAμM^−1^ cm^−2^	-	279 nM	AA, UA	6 days	[90]
	DPV	Ti_3_C_2_T_x_-MXene	SPCE	-	0.0134 μA nM^−1^	40–500 nM	1.3 nM	EP, NE, ST	15 days	[91]
DA	Amperometry	PtNi@N-GQDs	SPCE	3 s	0.279 μAμM^−1^ cm^−2^	0.0125–952 μM	0.005 μM	AA, UA, Glu, L-cys	25 days	[92]
	DPV	NiTsPc-ZnONPs-CNT	PGE	-	10 μA μM^−1^	-	7 nM	AA, UA, ST	-	[93]
	DPV,	GO-AgNPs@MWCNTs	GCE	120 s	-	0.5–6.5 μM	2.58 nM	AA, UA	20 days	[94]
	DPV	MoTe_2_/NC	GCE	-	0.814 μA μM^−1^	100 nM–50 μM	7.8 nM	UA, AA	16 days	[95]
	CV, EIS, DPV	MIP/4-MPBA/AuNPs	ANE	-	-	0.5 μM-1 mM	0.14 μM	AA, UA, Glu	10 days	[96]
	DPV, CA	MXene/DODA	ITO	-	4.098 μA μM^−1^	-	36.8 nM	UA, AA, Glu, Na⁺, K⁺, Ca^2+^, Mg^2+^	9 days	[97]
	DPV	CoS_2_-FeS_2_/HNCC	GCE	-	91.6 μAμM^−1^ cm^−2^	0.05–90 μM	9.3 nM	UA, AA, Glu, H_2_O_2_, Na⁺, K⁺	21 days	[98]
	CA	PCN-333 film	-	-	4637.78 μA mM^−1^ cm^−2^	0.5–140 μM	0.14 μM	AA	-	[99]
	CV, DPV, CA	pGr-MoS_2_	GCE	-	4.88 μA mM^−1^ cm^−2^	0.00001–10 μM	0.01 nM	AA, UA, EP, NE, Glu, ST	90 days	[100]
	SWV	Ce-MOF m-PdNFs-G4-MBs	GCE	-		10 pM–100 nM	6 pM	AA, BSA, Glu, L-cys	15 days	[101]
	CV	LINC	CPE		5.78 ± 0.32 nA nM^−1^	10 nM–1 μM	10 nM	-	-	[102]
	DPV	BNGrD	-	-	0.21 µA µM^−1^	0.5–500 µM	0.12 µM	Gl, UA, BSA, urea, Ala, Na^+^, K^+^	5 times, 48 days	[103]
	DPV	H-Mo-CoP/NC			11.89 μA mM^−1^ cm^−2^, 2.509 μA mM^−1^ cm^−2^	1 μM–50 μΜ, 50 μM–300 μM	55 nM	UA, AA, Gly, Glu, Na^+^, urea, SO_4_^2−^, EP, NE, GSH	30 times, 30 days	[104]
α-syn	DPV	-	GE	-	-	1 fM–10 pM	0.46 fM	AβO, AβF, IgG, L-cys, BSA, Glu, UA, DA	14 days	[78]
	SWV,	AuNPs/SWCNTs	SPCE	-	-	0.01–10 ng/mL	4.1 pg/mL	Aβ_42_, Aβ_40_	1 month	[77]
	LSV	SWCN, gold-nanourchin	IDE	-	-	-	1 fM	DJ-1	-	[75]
	EIS, DPV	-	FTO		133 μAng^−1^ mL	10–1000 ng/mL	3.62 ng/mL	Chol, BSA, GOX, LgG, DA, AA, UA	7 days	[79]
	EIS	-	GE	-	-	-	0.3 pg/mL	CRP, BSA	-	[105]
	EIS	-	LIG	5 s	-	-	-	-	7 h	[106]
	DPV	-	GE	-	-	1 fg/mL–0.1 ng/mL	0.57 fg/mL	AβO, AβF, IgG, L-cys	14 days	[107]
	CA	Ag ink	Paper-based electrode	-	-	0.002–128 ng/mL	0.002 ng/mL	-	disposable	[108]
	EIS	Au NPs	LIG	-	-	0.01–100 ng/mL	0.237 pg/mL	Tau, Aβ, BDNF	10 days	[109]
	EIS									
GFAP	DPV	rGO/PDA-MIP	-	10 min	-	1–10^6^ fg/mL	754.5 ag/mL	AA, UA, urea, vimentin, Gly, Glu	3 months	[81]
NfL	EIS	-	GE	-	-	-	5.21 ng/L	-	-	[82]
DJ-1	EIS	Au NPs	SPCE	-	0.167 kΩ nM^−1^	1–500 nM	1 nM	-	-	[83]

CV: cyclic voltammetry, DPV: differential pulse voltammetry, EIS: electrochemical impedance spectroscopy, LSV: linear sweep voltammetry, CA: chronoamperometry, FTO: fluorine-doped tin oxide electrode, IDE: integrated interdigitated electrode, ALIDEs: aluminum interdigitated electrode, ZTO: Zn_2_SnO_4_, MnO_2_ NSs: MnO_2_ nanosheets, S_v_: sulfur vacancy, TOC: 2,2,6,6 tetramethylpiperidine-1-oxyl (TEMPO)-oxidized cellulose nanofibers, CuAu SACs: CuAu single-atom catalysts, SPCE: screen-printed carbon electrode, CGC-500: congress grass carbon-500, MOF: metal–organic framework, Fe-HPSW-C: Fe-doped layered porous algal carbon, Fe/N-GR: disperse Fe-sites onto the N-doped graphene support, CDs: carbon dots, TEPA: tetraethy lenepentamine, MPTMS: (3-mercaptopropyl)trimethoxysilane, Yb_2_O_3_: ytterbium oxide, In_1_-N-C: indium anchored on nitrogen-doped carbon, PtNi@N-GQDs: platinum nickel bimetallic alloy nanoparticle-conjugated nitrogen-doped graphene quantum dots, SWCN: single-walled carbon nanotube, PGE: pyrolytic graphite electrode, PGE/NiTsPc-ZnONPs-CNT: a composite based on nickel phthalocyanine, zinc oxide nanoparticles, and carbon nanotubes, MoTe_2_/NC: N-doped carbon-supported molybdenum ditelluride, ANE: acupuncture needle electrode, MIP: molecular imprinted polymers, 4-MPBA: 4-mercaptophenylboronic acid, DODA: dimethyldioctadecylammonium bromide, HNCC: hollow nitrogen-doped carbon, PCN-333: porous Fe-based MOF, YO/BN: the nanocomposite of yttrium oxide decorated on boron nitride, Co_3_O_4_/SWCNTs-OH: cobalt oxide nanoparticles and hydroxylated single-walled carbon nanotubes composite, pGr-MoS_2_: a graphene-molybdenum disulphide nanocomposite, Ru-Ala-C_3_N_4_: single-atom ruthenium biomimetic enzyme, MB: methylene blue, m-PdNFs-G4-MBs: the surface modification of PdNF was utilized to increase the loading of MB through G-quadruplex-MB complexes, LIG: laser-induced graphene, LINC: laser-induced nanocarbon, CPE: carbonaceous printed electrodes, BNGrD: boron and nitrogen co-doped graphene-diamond, H-Mo-CoP/NC: hollow carbon nanocage/bamboo-like N-doped CNTs encapsulated with Mo-doped CoP nanoparticles, GSH: glutathione, Gly: glycine, GE: gold electrode. LIG: laser-induced graphene, rGO/PDA-MIP: reduced graphene oxide/polydopamine-molecularly imprinted polymer, AA: ascorbic acid, UA: uric acid, Glu: glucose, Gly: glycine, L-cys: L-cysteine, EP: epinephrin, NE: norepinephrine, ST: serotonin, BSA: bovine serum albumin, THAM: tromethamine, Thy: thymidine, Gl: glutamic acid, Ala: alanine, AβO: amyloid-β oligomer, AβF: amyloid-β fibers, IgG: immunoglobin G, Aβ_42_ and Aβ_40_: Alzheimer’s disease-associated proteins, DJ-1: proteins associated with Parkinson’s disease, Chol: cholesterol, GOX: glucose oxidase, CRP: C-reactive protein, Factor IX: clotting factor, Tau: tubulin-associated protein, Aβ: β-amyloid protein, BDNF: brain-derived neurotrophic factor.

### 3.3. Transistor Biosensors in PD Detection

A transistor sensor is a semiconductor device that regulates current flow and performs amplification and switching functions. It comprises three electrodes, namely the base, emitter, and collector. An appropriate voltage is applied between the base and the emitter to control the current flow between the collector and the emitter. Many researchers utilize oxide film as a channel layer and employ electron beam evaporation technology to fabricate the source and drain while adjusting sensitivity by varying gate voltage loading. The advantage of the sensor lies in its capability to detect multiple parameters through the utilization of physical quantities such as threshold voltage, material mobility, and conductivity of the channel layer, thereby enhancing accuracy and sensitivity.

#### 3.3.1. Dopamine Detection

**Figure 6:**
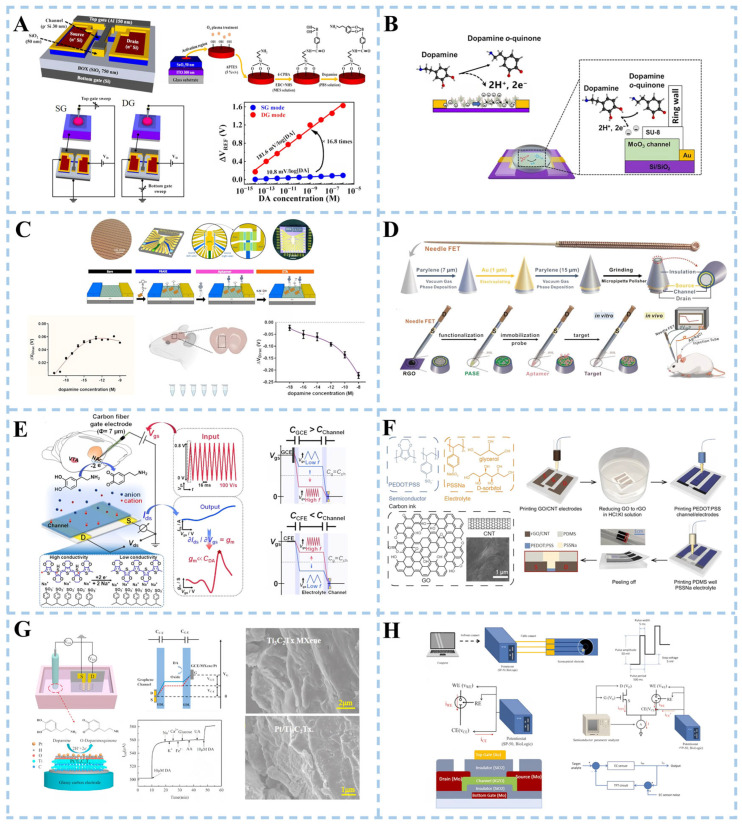
Schematic illustration of transistor biosensor for dopamine detection. (**A**) Double-gate structure field-effect transistor. Reproduced with permission: Copyright 2023, Multidisciplinary Digital Publishing Institute [110]. (**B**) Field-effect transistor sensor based on α-phase molybdenum oxide. Reproduced with permission: Copyright 2023, American Chemical Society [111]. (**C**) Graphene aptamer sensor. Reproduced with permission: Copyright 2022, BioMed Central Ltd. [113]. (**D**) Acupuncture needle-based transistor. Reproduced with permission: Copyright 2022, Wiley-VCH [112]. (**E**) Fast-scanning potential-gated organic electrochemical transistors. Reproduced with permission: Copyright 2022, Wiley-VCH [114]. (**F**) Fully 3D-printed organic electrochemical transistors. Reproduced with permission: Copyright 2023, Nature Portfolio [115]. (**G**) Pt/Ti_3_C_2_Tx MXene-based graphene electrochemical transistor. Reproduced with permission: Copyright 2022, Elsevier [116]. (**H**) Electrochemical sensor integrated with a feedback-loop indium-gallium-zinc oxide thin-film transistor. Reproduced with permission: Copyright 2023, Elsevier [117].

A transistor sensor consists primarily of a source, gate, drain, and channel. The researchers detected the electrical signal of the DA by applying voltage to the gate. Transistor sensors exhibit significant amplification effects on DA detection. Numerous studies focused on enhancing DA detection performance through modifications in the gate or channel doping. Hyun et al. [110] introduced a double-gate structure field-effect transistor (DGFET), which utilized dual-gate capacitive coupling for precise self-amplification of minute analyte potentials (Figure 6A). The biosensor platform consisted of a dopamine-sensitive extended gate (EG) sensing unit and a DG FET transducer unit. In the dual-gate configuration, the bottom-gate electrode operated the FET device by applying a gate voltage, while the top-gate electrode was connected to the EG sensing unit to receive the electrochemical potential of DA. The EG sensing unit was fabricated with glass as the substrate, while the ITO conductive layer and SnO_2_ sensing layer were prepared using RF magnetron sputtering technology. The ITO conductive layer was connected to the top-gate electrode of the DG FET via a cable. The surface potential of DA was transferred from the SnO_2_ sensing layer to both the ITO conducting layer and the top-gate electrode of the sensor unit. Experimental results demonstrate that in dual-gate (DG) mode, the sensitivity for DA detection is approximately 17 times higher compared to the single-gate (SG) mode. Tran et al. [111] reported a field-effect transistor sensor based on α-phase molybdenum oxide (α-MoO_3_) (Figure 6B). Molybdenum oxide (MoO_3_) is a material with special electronic and chemical properties, and its properties can be changed by adjusting its composition and structure. The layered structure of α-MoO_3_, along with its intercalation reactions associated with the oxidation/reduction in Mo ions, rendered the material highly promising for applications in neuromorphic computation. The interaction between DA and MoO_3_ led to a direct and localized charge transfer on the surface of MoO_3_, facilitating the detection of DA. This charge transfer altered the conductance of MoO_3_ (the ability of electrons to flow), thus affecting the output signal of the sensor.

Graphene has been widely reported as a common channel modification material. For the fabrication of in vivo FET devices in addition to good performance, the size of the device needs to be small enough to be inserted into the body as minimally invasively as possible [112]. Abrantes et al. [113] utilized a high-yield scalable approach optimized at the wafer level to integrate multiple graphene transistors on a small-sized chip (4.5 × 4.5 mm), namely, a multi-transistor array based on graphene (gMTAs) (Figure 6C). The sensor achieved a remarkably low detection limit of 1 aM for DA. The gMTAs platform, which could detect subtle changes in dopamine concentrations in small working volume samples of cerebral spinal fluid obtained from a mouse model of PD, is a powerful tool for studying Parkinson’s disease. Another study developed field-effect transistor sensors based on acupuncture needles used in medical applications [112] (Figure 6D). The needle body was sequentially coated with insulating layers of parylene, gold (Au), and parylene. Subsequently, the needle tip was polished to expose the structure of the inner layer, consisting of the stainless parylene-Au-parylene layer. A gold film serves as the source electrode, while stainless steel functions as the drain electrode. Reduced graphene oxide (rGO) drops were applied on the cross-section of the needle tip to create a modified channel connecting the source and drain electrodes. DA detection was achieved by further modifying with aptamers for specific recognition of DA molecules. This sensor has been successfully utilized for real-time monitoring of DA levels in normal rat brains. Its capability was also demonstrated to monitor changes in DA levels in a rat model of Parkinson’s disease. Furthermore, the sensor exhibited potential for detecting electrochemically inactive signaling molecules despite its limitation due to difficulties associated with functionalizing its small tip.

Electrochemical transistor sensors are a type of sensor that utilize the electrochemical principle to detect changes in current or potential, possessing dual characteristics of both electrochemistry and transistors. The sensors typically consist of electrodes, electrolytes, and transistor circuits. Their working principle involves converting the measured substance into electrical signals through electrochemical reactions before processing them with the transistor circuit. Depending on their channel materials, these sensors can be classified as organic or inorganic electrochemical transistor sensors.

The working principle of an organic electrochemical transistor is to control the opening and closing state of the transistor by controlling the potential change between the electrodes. This enables signal amplification, switching, and conversion functions. Li et al. [114] developed a sensor based on an organic electrochemical transistor (OECT) (Figure 6E). An interdigital electrode (IDE) of the sensor modified with poly(3,4-ethylenedioxythiophene)-poly(styrenesulfonate) (PEDOT: PSS) was used as the semiconducting channel. The sensor operated in rapid-sweep potential (FSP) gating mode, combining fast-sweep cyclic voltammetry (FSCV) selectivity with high OECT sensitivity. The detection limit for DA achieved by the sensor reached nanomolar levels. Massetti et al. [115] opted for PEDOT: PSS as the material for the OECT channel to present a fully 3D-printed OECT device (Figure 6F). The realization of printable functional inks for all components of the OECT (source, drain, gate, semiconductor channel, insulator, gate electrolyte, and substrate) embodied the concept of a completely 3D-printed OECT system. D-sorbitol ion reservoirs were capable of retaining mobile ions within the PEDOT: PSS electrolyzer, rendering it suitable as a plasticizer. A PDMS insulation layer was printed on the PEDOT: PSS channel and gate to define the trap of the electrolyte. This helped to better control the interaction between the electrolyte and the electrode. Additionally, a hydrogel was printed to increase ionic conductivity by introducing more ion species. The 3D-printed OECT demonstrated good DA biosensing capabilities.

The working principle of the inorganic electrochemical transistor sensor is based on the conductivity change in the semiconductor. The target substance reacts electrochemically with the semiconductor material, and electrons are produced or consumed, resulting in a change in electrical conductivity. Zhou et al. [116] proposed an electrochemical transistor sensor that utilized Ti_3_C_2_T_x_ MXene nanoparticles and a Pt nanoparticle-modified glassy carbon electrode (GCE) as the gate, with a single-layer graphene serving as the channel (Figure 6G). The Ti_3_C_2_T_x_ MXene showed a two-dimensional layered structure, characterized by an extensive specific surface area. The good selectivity of the sensor was attributed to the hydrophilic negative charge of Ti_3_C_2_T_x_ MXene. This property enabled it to exhibit electrostatic adsorption towards protonated DA and electrostatic repulsion towards negatively charged interfering substances. The good catalytic performance of Pt nanoparticles promoted the electrochemical reaction of DA.

The working principle of thin-film transistors (TFT) is based on controlling the electronic conductive properties and field effects of semiconductor materials. Specifically, the core part of the TFT consists of an insulating layer covered with source, drain, and gate structures. Between the source and drain, a semiconductor film acts as a channel. When no voltage is applied to the gate, electrons in the semiconductor film are in a free state and float on the film. In this case, electrons can flow freely, making the TFT electrically conductive. However, when a specific voltage is applied to the gate, the electric field generated by it changes the band structure of the semiconductor material creating a barrier that affects electron flow. The flow of electrons is controlled by changing gate voltage to realize functions such as signal amplification, switching, and logic operation. A novel biosensor for DA detection has been developed by combining a thin-film transistor with an electrochemical biosensor. The new sensor consists of a typical electrochemical biosensor, a TFT, and a constant current source [117] (Figure 6H). The electrochemical sensor was equipped with a Nafion-modified screen-printed carbon electrode. The electrochemical sensor and the TFT formed differential pairs in the circuit to provide negative feedback control along with the constant current source. The differential pair and negative feedback control design could effectively suppress the fluctuation of the electrochemical sensing signal, thereby improving the stability and reliability of the DA REDOX sensing signal.

#### 3.3.2. α-Synuclein Detection

**Figure 7:**
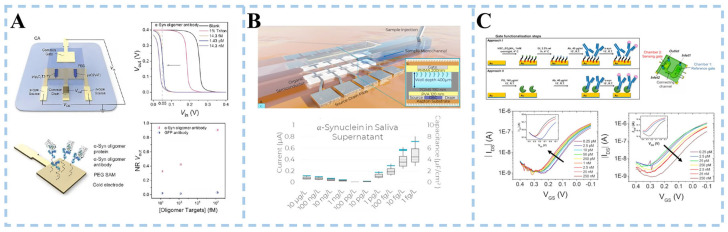
Schematic illustration of transistor biosensor for α-synuclein detection. (**A**) N-type organic electrochemical transistor. Reproduced with permission: Copyright 2023, Wiley-VCH GmbH [118]. (**B**) Organic electrolyte-gated FET aptasensor. Reproduced with permission: Copyright 2023, American Chemical Society [119]. (**C**) Coplanar electrolyte-gated organic field-effect transistor. Reproduced with permission: Copyright 2020, Elsevier [120].

OECT detects the presence of α-syn by monitoring changes in its electrical conductivity. When α-syn binds to the channel material, the transfer curve of OECT shifts, indicating significant changes in electrical signals. This change can be used for quantitative analysis of α-syn concentration and detection. Wang et al. [118] utilized gold as a gate electrode to achieve high sensitivity in detecting α-syn by connecting two OECT channels, which is equivalent to constructing a complementary amplifier (CA). The two channel-modified materials were lactone N-type conjugated polymer p(C2F-V) and P-type polymer p(g_3_C_2_T2-T), synthesized through aldol polymerization (Figure 7A). The CA transfer curve of α-synuclein oligomer exposure was significantly skewed under appropriate bias conditions. The transfer curve of the OECT shifted and the electrical signal changed noticeably. Based on this, the concentration of α-syn was analyzed, and its detection was achieved.

Massey et al. [119] demonstrated an organic electrolyte-gated field-effect transistor (OEGFET) aptasensor capable of quantifying levels of α-Syn aggregation in saliva (Figure 7B). The traditional electrolyte-gated field-effect transistor has the organic semiconductor (OSC) layer in direct contact with the electrolyte sample. Signal magnitude is achieved by increasing charge injection and reducing signal attenuation. However, it degrades OSC and introduces noise through pseudocapacitance effects. The aptasensor reported minimizes the integration challenges of traditional electrolyte-gated OFETs by physically isolating the gate and OSC from the electrolyte using ultra-thin polymer dielectric layers. Small single-stranded DNA molecules generated from in vitro selection were used as aptamers. The low detection limit of the α-Syn monomer in saliva supernatant samples by the OEGFET sensor covered the physiological range of α-Syn in saliva.

Ricci et al. [120] designed a sensor based on a dual coplanar electrolyte-gated organic field-effect transistor in a microfluidic chamber (Figure 7C). The gate electrode was functionalized with a monoclonal anti-(α-synuclein) antibody. Two different surface functionalization strategies were employed, including an amino-terminated self-assembled monolayer activated by glutaraldehyde and the His-tagged recombinant protein G. Both strategies showed similar sensitivities and LOD values, with a linear range of 0.25 pM–25 nM for each strategy. Cross-contamination between the organic semiconductor and the sensing gate was prevented by the design of the microfluidics arrangement. In addition, details of the different transistor biosensing for DA and α-syn detection are shown in Table 3.

#### 3.3.3. Other Biomarker Detection

Neurofilaments are structural scaffolding proteins of the neuronal cytoskeleton. Upon axonal injury, the neurofilament light chain (NfL) is released into the interstitial fluid and eventually reaches the cerebrospinal fluid and blood. In recent years, NfL has emerged as a potential biomarker for the neurological disorder Parkinson’s disease. Solodka et al. [121] developed an organic field-effect transistor sensor for NfL detection by immobilizing anti-NfL antibodies on a gate electrode. The sensor can quantify NfL to the pM level. The effectiveness and repeatability of the sensor for detecting NfL in buffer solution at physiological PH were verified by a large number of independent experiments.

**Table 3:** Different transistor sensing schemes for Parkinson’s disease biomarker detection.

Target	Type	Nanoparticles	Response Time	Sensitivity	Linear Range	LOD	Selectivity	Stability	Ref
DA	FET	rGO	A few seconds	-	1 nM–10 μM	370 pM	Gl, AA, Ach, EP, NE, K^+^	10 times, 7 days	[112]
	AC-SGT	Graphene	Second	-	3 nM–300 μM	3 nM	AA, UA	30 days	[122]
	OECT	rGO/CNT	-	-	1–100 μM	6 μM	-	-	[115]
	FET	MoO_3_	1 s	-	-	100 nM	K^+^, Na^+^, Ca^2+^	-	[111]
	TFT	-	-	2.91 × 10^−2^ μA/μM	2–50 μM	0.486 μM	UA, AA	-	[117]
	FET	-	-	373.98 mV/log(DA)	10 fM–1 μM	10 fM	-	-	[110]
	FET	MUA-AuNCs	-	4200 nA/μM	0.03–5 μM	0.01 μM	AA, UA, Gl, Asp, Gly, Na^+^	3 times, 90 min	[123]
	IECT	Pt/Ti_3_C_2_T_x_ MXene, graphene	-	-	50 nM–10 μM, 10 μM–9 mM	50 nM	Glu, Na^+^, K^+^, Ca^2+^, Fe^2+^	15 days	[116]
	OECT	-	Real-time	0.899 s/M	-	5 nM	DOPAC, UA, AA	6 times	[114]
	FET	Graphene	-	-	-	1 aM	AA, L-DOPA, L-Tyr	-	[113]
	FET	Fe_3_O_4_@AuNPs	-	3.16 mA/μM	-	3.3 nM	DOPA, NE, 4-PC, 3,4-DHBA, EP, GA	15 times, 10 days	[124]
	FET	AuNPs/graphene	Real-time	12.23 mV/decade	1 × 10^−19^–1 × 10^−11^ M	6 × 10^−20^ M	Glu, UA, AA	-	[125]
	OECT	-	-	-	1 nM–100 μM	61 nM	AA, UA	500 times	[126]
	FET	-	-	10.69 mV/log(DA)	1 fM–1 nM	0.523 fM	UA, AA	-	[127]
	OECT	CNT/Pt NPs	Real-time	-	-	5 nM	UA, AA, DOPAC	7 days	[128]
α-syn	OECT	-	-	-	14 fM–14 nM	fM level	-	-	[118]
	FET	-	-	-	100 fg/L–10 μg/L	10 fg/L	-	15 days	[119]
	FET	-	-	37 (±5) mV/decade	0.25 pM–25 nM	0.25 pM	-	-	[120]
NfL	FET	-	A few minutes	-	100 fM–10 nM	30 fM	-	-	[121]

FET: field-effect transistor, AC-SGT: all-carbon solution-gated transistor, OECT: organic electrochemical transistor, IECT: inorganic electrochemical transistor, TFT: thin-film transistor, MoO_3_: molybdenum oxide, MUA-AuNCs: 11-mercaptoundecanoic acid-gold nanoclusters, poly-SiNW: poly-crystalline silicon nanowire, CNT: carbon nanotube, Gl: glutamate, AA: ascorbic acid, Ach: acetylcholine, EP: epinephrine, NE: norepinephrine, Asp: aspartic acid, Gly: glycine, Glu: glucose, DOPAC: 3,4-dihydroxyphenylacetic acid, L-DOPA: 3,4-Dihydroxyl-L-phenylalaline, L-Tyr: L-Tyrosine, 4-PC: 4-propylcatechol, 3,4-DHBA: 3,4-dihydroxybenzoic acid, GA: gallic acid.

## 4. Conclusions

With the progressive increase in the average age of the global population, there is an inevitable surge anticipated in the number of individuals affected by PD. The pathophysiology and pathogenesis of PD remain incompletely defined, with the current clinical signs and symptoms only manifesting in the advanced stage of the disease. Therapeutic intervention at this stage merely alleviates these symptoms without necessarily inhibiting the underlying pathological pathway. For instance, levodopa effectively alleviates motor symptoms in patients by replenishing depleted dopamine levels, but it is also accompanied by side effects such as gastrointestinal discomfort. Consequently, there exists an urgent and unprecedented need to develop cutting-edge technologies that can offer sensitive, rapid, reliable, and cost-effective methods for PD detection based on emerging analytical techniques. In this comprehensive review, we provide a concise overview of recent advancements in this field while highlighting the remarkable pace at which biosensors utilizing optical, electrochemical, and transistor methodologies have evolved.

Electrochemical biosensors utilize the electrochemical properties of PD-related analytes such as dopamine and α-synuclein to detect their presence in biological samples. These biosensors offer advantages such as high sensitivity, portability, and real-time monitoring. Optical biosensors employ the interaction between light and PD biomarkers to detect their presence. These biosensors utilize fluorescence, absorbance, or surface plasmon resonance techniques for detection and offer advantages such as high specificity, label-free detection, and multiplexing capabilities. Transistor sensors, based on specific binding reactions between biometric elements and target biomarkers, detect biomarkers by capturing and converting changes in physical or chemical signals caused by such reactions into electrical signals. This method has high specificity and sensitivity. However, despite the extensive progress summarized in this paper, the practical application of sensors based on PD biomarker detection still faces several key challenges that urgently need to be addressed (Table 4):

For optical sensors, optical signals are easily affected by environmental factors such as temperature, humidity, and light, and the stability of optical biosensors needs to be further improved in the future to reduce the interference of environmental factors. On the other hand, optical biosensors often require the use of sophisticated optical instruments and equipment, increasing the complexity and cost of the system. The reduction in the cost of optical instruments and equipment would make them easier to promote and apply.

Electrochemical biosensors are susceptible to interference from electroactive substances in solution. New electrode materials and signal processing technology are developed to improve the anti-interference ability of electrochemical sensors. After the sensor is used for a long time, the stability and reproducibility of the electrode are easily affected. Research on methods to improve electrode stability and reproducibility and extend the service life of sensors is also still in progress and must be expanded in the future.

Compared with the previous two kinds of biosensors, the technological maturity of transistor biosensors (especially field-effect transistor biosensors) is relatively low, and further research and development are needed. Currently, the channel layer materials used to prepare FET biosensors are limited, which limits the application range of sensors. The design and performance optimization of channel materials should be considered.

With regard to biosensors based on PD biomarker detection, most of the progress and experimental research has been limited to laboratory settings, showing a huge gap in practical applications. In addition, the transition to practical applications requires attention to scalability, cost-effectiveness, and eco-friendliness, critical for mass production and widespread adoption. Furthermore, PD overlaps with the features of some other diseases, such as multiple-system atrophy, especially in the early stages of the disease, so there is still a need to improve detection specificity.

In addition to addressing these questions, future research should prioritize the exploration of novel biomarkers and advanced sensing technologies to achieve more accurate, reliable, and timely diagnosis of Parkinson’s disease (PD). Emerging sensor platforms exhibiting transformative potential include the following: 1. Nanopore sensors. Nanopore-based sensors demonstrate unique capabilities in single-molecule resolution analysis of pathological α-synuclein isoforms; 2. Exosome detection sensors. Exosome detection sensors targeting neuron-derived extracellular vesicles enable non-invasive interrogation of central nervous system biomarkers in peripheral biofluids; 3. Microfluidic integrated sensors. Microfluidic-integrated sensors significantly enhance the sensitivity of exosomal biomarker detection. The miniaturized systems allow automated isolation and analysis of exosomes, which carry disease-specific molecular signatures. Integrating these technologies with AI-driven data analysis may ultimately enable multiplexed, point-of-care platforms for early PD screening and progression monitoring.

## Figures and Tables

**Table 4:** Comparison of advantages and challenges of Parkinson’s disease biomarker detection with different sensors.

Sensor Type	Advantages	Challenges
Optics	● High sensitivity	● Improve stability
	● Non-contact detection	● Reduce cost
	● Multichannel detection	
Electrochemistry	● High sensitivity	● Improve anti-interference ability
	● Real-time monitoring	● Extend electrode life
	● Simple operation	
Transistor	● High sensitivity	● Improve technology maturity
	● High integration	● Expand the scope of application
	● Real-time monitoring	

## Data Availability

Not applicable.
